# High-efficiency tri-band quasi-continuous phase gradient metamaterials based on spoof surface plasmon polaritons

**DOI:** 10.1038/srep40727

**Published:** 2017-01-12

**Authors:** Yongfeng Li, Hua Ma, Jiafu Wang, Yongqiang Pang, Qiqi Zheng, Hongya Chen, Yajuan Han, Jieqiu Zhang, Shaobo Qu

**Affiliations:** 1 College of Science, Air Force Engineering University, Xi,an 710051, People’s Republic of China

## Abstract

A high-efficiency tri-band quasi-continuous phase gradient metamaterial is designed and demonstrated based on spoof surface plasmon polaritons (SSPPs). High-efficiency polarizaiton conversion transmission is firstly achieved via tailoring phase differece between the transmisive SSPP and the space wave in orthogonal directions. As an example, a tri-band circular-to-circular (CTC) polarization conversion metamateiral (PCM) was designed by a nonlinearly dispersive phase difference. Using such PCM unit cell, a tri-band quasi-continuous phase gradient metamaterial (PGM) was then realized by virtue of the Pancharatnam-Berry phase. The distribution of the cross-polarization transmission phase along the *x*-direction is continuous except for two infinitely small intervals near the phases 0° and 360°, and thus the phase gradient has definition at any point along the *x*-direction. The simulated normalized polarization conversion transmission spectrums together with the electric field distributions for circularly polarized wave and linearly polarized wave demonstrated the high-efficiency anomalous refraction of the quasi-continuous PGM. The experimental verification for the linearly polarized incidence was also provided.

As for conventional optical elements such as mirror, prisms, lens, wave plates, spiral phase plates, holograms as well as some diffractive elements, the phase modulations are accumulated by the optical path difference achieved via designing both the geometries and the refractive index profile. Therefore, the thicknesses of the optical elements are usually larger than or comparable to the incident wavelength. With the rapid development of electromagnetic metamaterials, the polarization manipulations can also be achieved using anisotropic or chiral metamaterials^1^,^2^, yet still with thickness limitations for broadband or wideband use. In this regard, the concept of phase “discontinuities” is proposed quite recently. Electric/Magnetic resonators and polarization converters with sub-wavelength sizes can usually provide an abrupt phase change to the co- and cross-polarization reflection/transmission, respectively. Accordingly, an array of these sub-wavelength unit cells with spatially varying phase responses can be used to control the wave-fronts with much more degrees of freedom. These artificial sub-wavelength unit-cell-arrays are named as the phase gradient metamaterial (PGM)^3^,^4^,^5^,^6^,^7^,^8^,^9^,^10^,^11^,^12^,^13^,^14^,^15^,^16^,^17^,^18^,^19^,^20^. By virtue of the PGM, a lot of optical functions can be realized including anomalous reflection/refraction^4^,^5^,^6^,^7^,^8^,^9^,^10^, optical focusing^11^,^12^,^13^,^14^, polarization conversion^15^,^16^,^17^, surface wave excitation^18^,^19^,^20^, beam splitting and imaging.

Surface plasmon polaritons (SPPs) are collective oscillations of free electrons trapped at metal-dielectric interfaces^21^. Natural SPPs can’t exist in the microwave frequency regime, but can be supported by the plasmonic metamaterials named as spoof surface plasmon polaritons (SSPPs). The plasmonic metamaterials are usually generated by decorating periodic arrays of sub-wavelength grooves, holes, or blocks on the metal surface, which have been proposed to realize the SSPPs at terahertz and microwave frequencies^22^,^23^,^24^,^25^,^26^. Owing to the deep sub-wavelength characteristic of the SSPP, the continuous phase accumulation of the SSPP can be approximately considered as “discontinuous” compared with the space wave. By designing the *k*-dispersion of the SSPP, the phase accumulation at a fixed distance can be manipulated freely. In our previous works, a high-efficiency dispersive PGM was achieved based on the SSPP coupling based transmissions^27^.

In this paper, a tri-band quasi-continuous PGM^28^ based on the SSPP coupling is proposed and experimentally demonstrated. The planar plasmonic structure consisting of corrugated metallic strips was employed to couple and guide the SSPP. Via aligning the SSPP coupling based transmission and the free space wave transmission in two orthogonal directions, a tri-band circular-to-circular (CTC) polarization conversion metamaterial (PCM) was designed and demonstrated by simulations. Based on Pancharatnam-Berry (PB) phase, a tri-band quasi-continuous PGM was achieved by using the unit cells of the CTC PCM as the sub-unit cells. Under circularly polarized (CP) wave incidence, the transmitted wave was efficiently converted into the cross-polarized CP wave and anomalously refracted. Under linearly polarized (LP) wave incidence, the transmitted wave was divided into two beams of CP waves and anomalously refracted along two opposite directions due to the opposite cross-polarization transmission phase gradients for left-handed circularly polarized (LCP) and right-handed circularly polarized (RCP) incidence. Both the simulated and measured normal transmittivity, as well as normalized polarization conversion transmission spectrum demonstrated the high-efficiency anomalous refraction of the tri-band quasi-continuous PGM.

## Result

### SSPP mediated by plasmonic structure consisting of corrugated metallic strips

**Figure 1:**
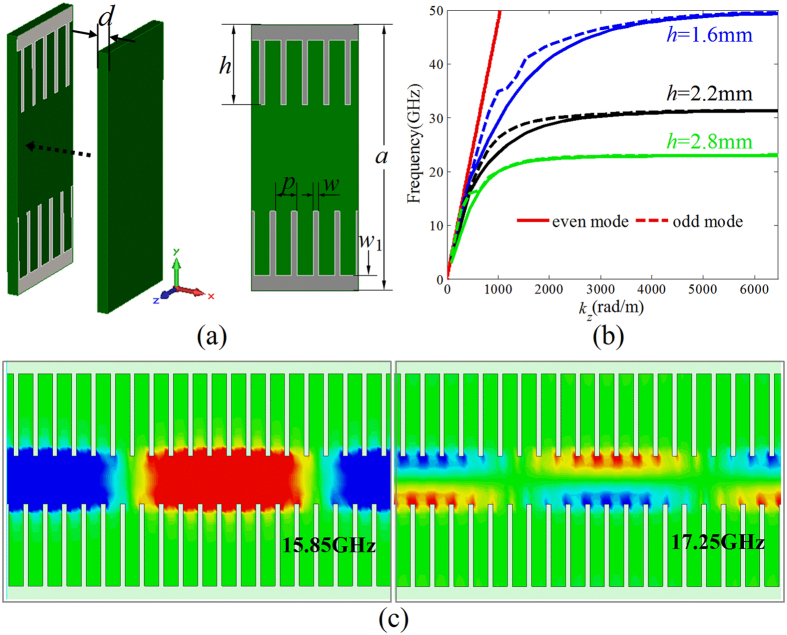
SSPP mediated by the plasmonic structure consisting of corrugated metallic strips. (**a**) The structural composition of the planar plasmonic structure. (**b**) Dispersion relations of the SSPP supported by the plasmonic structures with different strip length *h* = 1.6 mm, 2.2 mm, and 2.8 mm. (**c**) Distributions of the electric field component *E*_*y*_ in the *y*-*z* plane for odd and even modes at different frequencies (15.85 and 17.25 GHz) on the plasmonic structure with *h* = 2.2 mm.

As shown in Fig. 1, the planar plasmonic structure consisting of corrugated metallic strips is proposed to mediate the SSPP at microwave frequencies. The corrugated metallic strips are etched on the 0.25 mm-thick F4B (*ε*_*r*_ = 2.2, tan*δ* = 0.001) dielectric substrate, which is covered with another 0.25-thick F4B dielectric substrate. The geometrical parameters are designed to be: *a* = 6 mm, *d* = 0.25 mm, *p* = 0.48 mm, *w* = 0.12 mm, and *w*_1_ = 0.35 mm. In order to analyze the SSPPs mediated by the plasmonic structures, the simulated dispersion diagrams of the *y*-polarized waves on the plasmonic structure with different strip length *h* (1.6 mm, 2.2 mm, and 2.8 mm) are given in Fig. 1(b). The red solid line is the dispersion relationship of the light line. It can be found that all the dispersion relations lie below the light line. The propagation constant *k*_*z*_ for the *y*-polarized wave on the plasmonic structure is much larger than that of the wave in the free space. Thus the *y*-polarized wave propagating on the plasmonic structure can be considered as the SSPPs in GHz frequency regime, which are confined and enhanced within the sub-wavelength regions. This can be demonstrated by the simulated electric field modes given in Fig. 1(c). It is found that the electric fields of the SSPP are highly confined on the blade structure. The wavelength of the SSPP observed from the distributions of the electric fields is highly reduced compared with the wavelength in the free space. Obviously, the dispersion curves include two branches corresponding to the even and odd modes, respectively, and the asymptotic frequencies of two modes are quite close to each other. This also can be demonstrated by the simulated electric field mode component *E*_*y*_ at 15.85 GHz and 17.25 GHz, respectively, corresponding to the odd and even modes. Additionally, the propagation mode will be cut off as the frequency is greater than the asymptotic frequency. The asymptotic frequencies and the propagation constant at a fixed frequency can all be easily tailored by changing the metallic strip length *h*. In detail, the propagation constant of the SSPP increases with increasing strip length *h*, and the asymptotic frequency moves toward the lower frequency as the strip length *h* is increased.

### Tri-band PCM based on SSPP coupling

**Figure 2:**
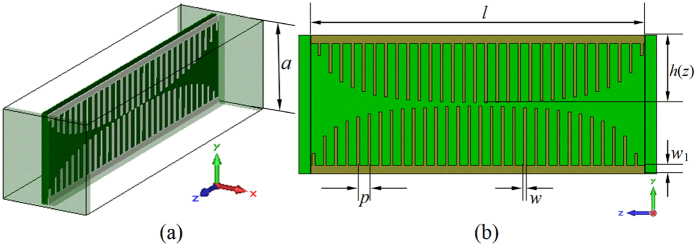
Schematic pictures for the unit cell of the tri-band CTC PCM. (**a**) Perspective view of the PCM unit cell. (**b**) Front view of the plasmonic structure consisting of corrugated metal strips with modulated length *h*(z).

The unit cell of the PCM is illustrated in Fig. 2, where Fig. 2(a) gives the perspective view, and Fig. 2(b) the front view of the plasmonic structure consistng of modulated metallic strip length by a gradient propagation constant for the mediated SSPP. The planar plasmonic structure placed in *yoz* plane consists of 30 metallic strips etched between two 0.5 mm-thick F4B (*ε*_*r*_ = 2.2, tan*δ* = 0.001) dielectric substrates is used to couple and guide the SSPP. The repetition periods of the unit cell in *x*- and *y*-directions are *a* = 6 mm and *b* = 6 mm. The total length of the planar plasmonic structure along *z-*direction is *l = *14.4 mm. The spatial distribution of the metallic strip length *h(z*) for the planar plasmonic structure is modulated according to the spatial distribution of the SSPP propagation constant *k*_spp_(z), which linearly grows from the smallest value to the highest value firstly, and then drops back to the smallest value linearly. This specific design can guarantee a high conversion efficiency between the SSPP and free space wave due to the wave vector matching at the air-dielectric and dielectric-air interfaces. Thus the transmission can be greatly enhanced.

**Figure 3:**
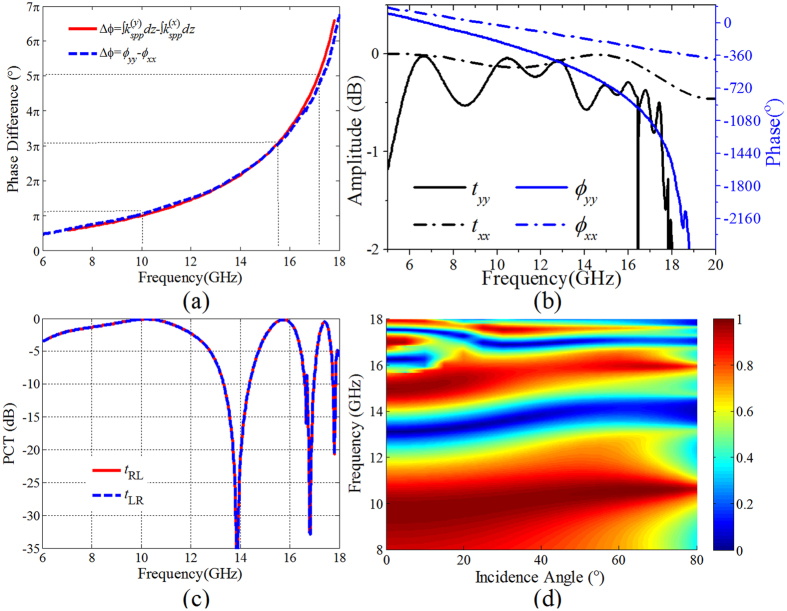
Simulation and analysis of the tri-band CTC PCM. (**a**) The phase difference calculated from the simulated co-polarization transmissions under *y*- and *x-*polarized wave normal incidence as well as from the dispersion of the SSPP on the plasmonic structure. (**b**) Transmittivities and transmission phases for *y*- and *x*-polarized wave normal incidence from z-direction, respectively. (**c**) Simulated CTC polarization conversion transmittivities (PCT) for LCP and RCP waves normal incidence onto the designed PCM. (**d**) Simulated amplitude of the CTC polarization conversion transmission under LCP wave incidence with the incidence angle changed from 0° to 80°.

**Figure 4:**
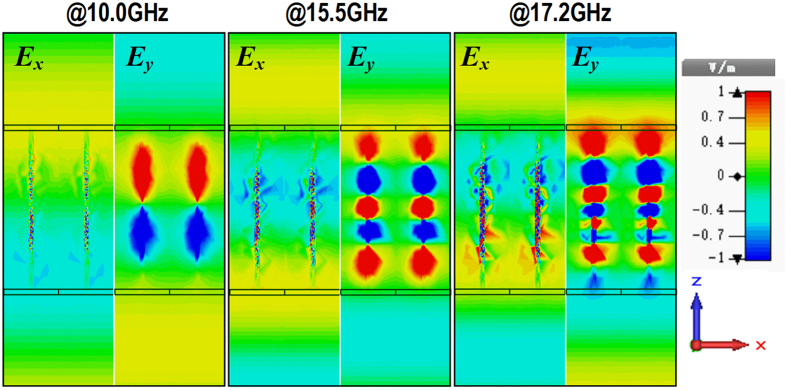
Distributions of the electric field *x*- and *y*-components (*E*x and E_*y*_) in *xoz*-plane under LCP wave normal incidence from +z direction.

Due to the nearly perfect SSPP coupling on the planar plasmonic structure, the *y*-polarized incidence wave is converted into SSPP mode with larger *k* while the *x*-polarized incidence wave is kept as propagating mode in the free space. Hence, a larger phase accumulation can be obtained for the *y*-polarized transmitted wave. This leads to a phase difference between the two orthogonal components of transmitted wave, a necessary condition for the polarization conversion. The phase difference between the transmitted *x*- and *y-*polarized waves can be expressed as


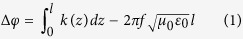


where *f* is the working frequency, *k*(z) the spatial distribution of the propagation constant for the mediated SSPP, and *l* the length of the plasmonic structure. As an example, the phase difference was designed to be π at the working frequency 10 GHz by designing the spatial distribution of *k(z*). Due to the nonlinear dispersion of the SSPP, this phase difference increases with increased frequency. As shown in Fig. 3(a), the phase difference versus frequency calculated according to the dispersion of the mediated SSPP is given using the red line, and the simulated phase difference between the co-polarization transmissions under *y*- and *x*-polarized wave normal incidence is given by the blue line. From the figure, it is observed that the simulated phase difference is in good accordance with the calculated result. The phase differences π, 3π, and 5π are corresponding to the frequencies 10.0, 15.5, and 17.2 GHz. Figure 3(b) gives the simulated amplitudes and phases of the co-polarization transmission coefficients for *y*- and *x*-polarized wave normal incidence. From the figure, we can find that the phase versus frequency is linear and nonlinear for *x-* and *y*-polarized wave incidence, respectively corresponding to the free space wave and SSPP. The co-polarization transmittivities under *x*- and *y*-polarized wave normal incidence are all greater than −1 dB over a wide frequency range from 5 GHz to 18 GHz. Assuming that the electric field of the incidence CP wave is expressed as


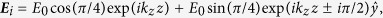


where “+” is for LCP and “−” for RCP. The transmitted field can be expressed as


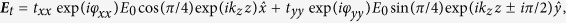


where *t*_*xx*_exp(*iφ*_*xx*_) and *t*_*yy*_exp(*iφ*_*yy*_) are the co-polarization transmission coefficients under *x*- and *y*-polarized waves incidence, respectively. According to previous analysis, the amplitudes of the transmission coefficients at the two orthogonal directions are approximately 1. Therefore, if the phase difference is π, the transmitted waves will be cross-polarized waves. Accordingly, high-efficiency circular-to-circular (CTC) polarization conversion will be achieved at the frequencies 10.0, 15.5, and 17.2 GHz. To verify the designed PCM, the CTC polarization conversion transmittivities (PCT) under LCP and RCP wave normal incidence are simulated and given in Fig. 3(c). It is found that this PCM can achieve high-efficiency CTC polarization conversion transmission in three bands. The CTC PCT is greater than −0.5 dB in frequency regimes: [8.5 10.8]GHz, [15.4 16.2]GHz, and [17.1 17.3]GHz. Considering the polarization conversion transmission under oblique incidence, the simulated amplitude of the cross-polarization transmission coefficient for LCP wave incidence with the incidence angle changed from 0° to 80° is shown in Fig. 3(d). From the figure, the CTC polarization conversion transmissions in all the three frequency bands have a blue shift by increasing the incidence angle. By contrast, the polarization conversion transmission is not sensitive at the first two frequency bands, and that is very sensitive at the third frequency band. Figure 4 shows the simulated distributions of the electric field *x*-, *y*-components (*E*_*x*_and *E*_*y*_) in the *xoz* plane for LCP wave normal incidence from +*z* direction at the frequencies 10.0 GHz, 15.5 GHz and 17.2 GHz. It is found that the phase accumulation differences between the electric field *y*- and *x*-components are π, 3π, and 5π, respectively, at the frequencies *f* = 10.0 GHz, 15.5 GHz and 17.2 GHz.

### Quasi-continuous PGM based on Pancharatnam-Berry (PB) phase

**Figure 5:**
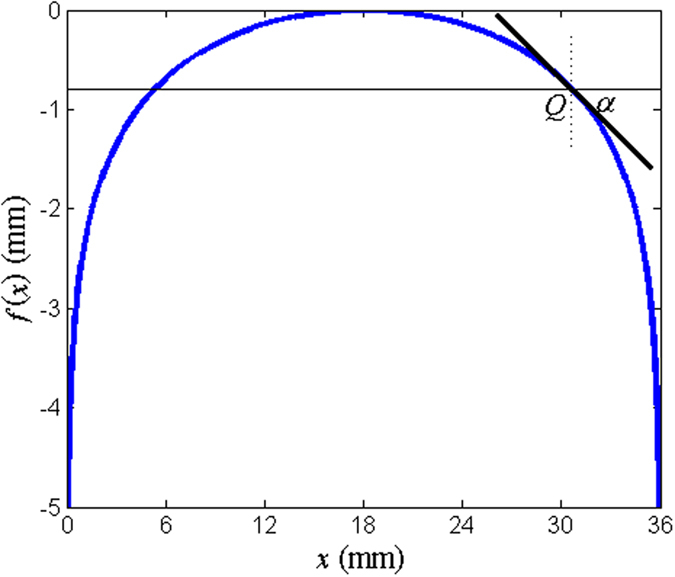
The curve plotted in line with function (2), where the parameters *a* = π/36, *b* = π/2, and *c* = 0.

Using the PCM designed above, the tri-band quasi-continuous PGM can be achieved based on PB phase. As shown in Fig. 5, the given curve is described using the following function,


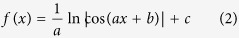


where *a, b* and *c* are arbitrary constants, and are chose to be *a* = π/36, *b* = −π/2, and *c* = 0 in this figure. Taking the derivative of this function with respect to *x*, we can derive that *f’(x*) = −tan(*ax* + *b*), which is the gradient of the tangent line at any point *Q* on this curve. Thus *α* = −*ax*-*b* is the corresponding angle, which is a linear function of *x*. The quasi-continuous tri-band PGM is gained by bending the planar plasmonic structure of the tri-band PCM according to the curve given in Fig. 5. For the wave polarized along the tangential direction incidence, the high-efficiency transmission is obtained based on the SSPP coupling. But for the wave polarized along the normal direction, the incidence wave is highly transmitted maintaining the free space wave. Accordingly, the high-efficiency CTC polarization conversion transmission can be achieved at any position on this curve. Via tailoring the PB phase, the phase shift of the cross-polarization transmission under CP wave incidence can be manipulated with much more degrees of freedom. In detail, the phase difference of the cross-polarization transmissions between *x*_1_ and *x*_2_ can be expressed as 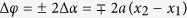. where “+” is for LCP wave incidence and “−” for RCP. Therefore, the realized phase gradient along *x-*direction is ▽*φ* = 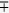
2*a*. Different from the general PGM, this phase gradient has definition at any *x*-coordinate except for *α* = ±π/2. The spatial distribution of the realized cross-polarization transmission phase about *x*-coordinate is quasi-continuous.

**Figure 6:**
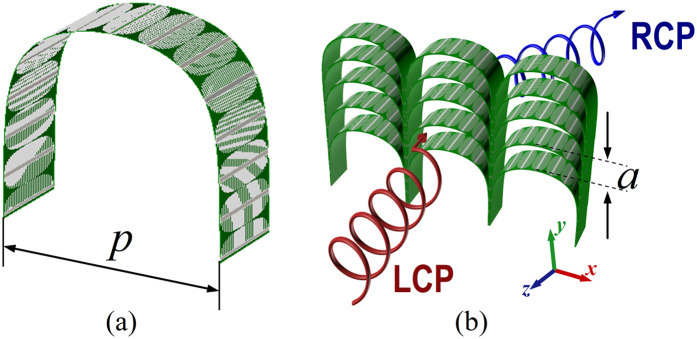
Structural views of the tri-band quasi-continuous PGM. (**a**) Perspective view of the curved plasmonic structure surface. (**b**) Perspective view of the designed tri-band quasi-continuous PGM consisting of a 5 × 3 array of super units.

**Figure 7:**
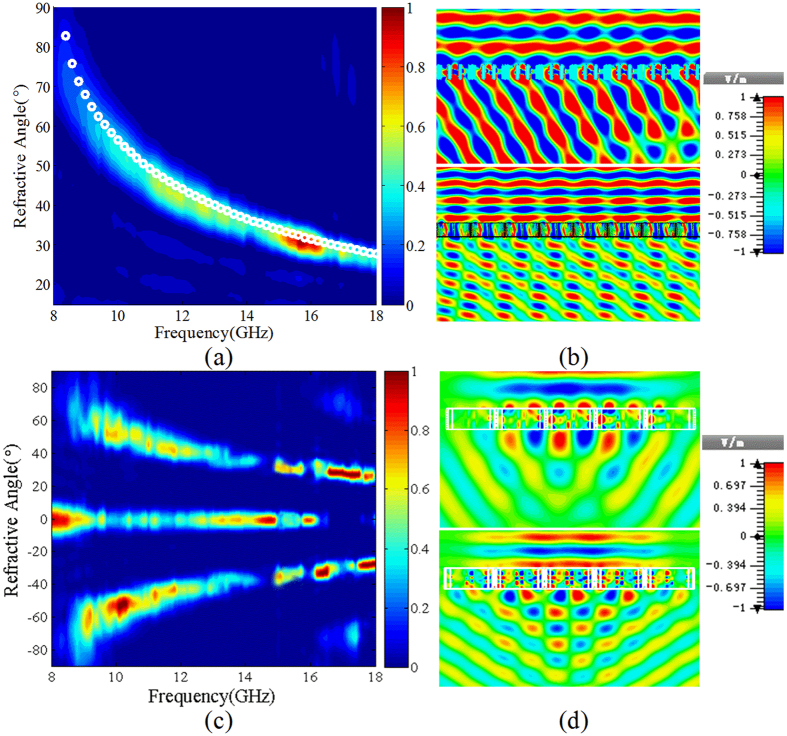
Simulated results for LCP wave and *x*-polarized wave normal incidence onto the tri-band quasi-continuous PGM. (**a**) The normalized cross-polarization transmission spectrum for LCP wave normal incidence. (**b**) Distributions of the electric field component *E*_*y*_ for LCP wave normal incidence at the frequencies *f* = 10 GHz and *f* = 15.5 GHz. (**c**) The normalized polarization conversion transmission spectrum for *x*-polarized wave normal incidence. (**d**) Distributions of the electric field component *E*_*y*_ for *x*-polarized wave normal incidence at the frequencies *f* = 10.0 GHz and *f* = 15.5 GHz.

Figure 6 shows the designed PGM consisting of a 5 × 3 array of super units, where Fig. 6(a) gives a super unit of the PGM, and Fig. 6(b) the perspective view of the PGM. The repetition periods of the super unit in *x*- and *y-*directions are *p* = 36 mm and *a* = 6 mm, respectively. The incidence CP wave will be converted into the cross-polarized wave with high efficiency and anomalously-refracted due to the cross-polarization transmission phase gradient. In addition, the phase gradients under LCP and RCP wave incidence have the same value but opposite signs, thus under the LP wave incidence, the transmitted wave will be decomposed into two beams of CP waves and anomalously refracted along opposite directions. To verify the anomalous cross-polarization transmissions of the designed PGM, full-wave numerical simulation is performed using CST Microwave Studio software to calculate the normalized anomalous transmission spectrum in the frequency range from 8 GHz to 18 GHz under LCP wave normal incidence from +z direction. The calculated result is shown in Fig. 7(a). In the figure, the *x*-coordinate labels the frequency of the incidence CP wave, and the *y*-coordinate denotes the refraction angle. The color in the figure represents the normalized cross-polarization transmittivity. The theoretically calculated refraction angle versus frequency is given in the figure marked by the white “o”. Obviously, the simulated anomalous transmission spectrum is well consisted with the theoretical refraction angle. The incidence LCP wave is highly refracted in three frequency bands of 8.5–13.5 GHz, 15.4–16.3 GHz, and 17.0–17.3 GHz. These frequency bands are exactly corresponding to the three polarization conversion frequency bands in Fig. 3, respectively. In the frequency range from 8 GHz to 8.33 GHz, the incidence LCP is highly transmitted and coupled into surface electromagnetic wave.

The distributions of the electric field components for LCP wave normal incidence from +z direction are simulated as depicted in Fig. 7(b), where the top and bottom figures are the *y*-components of the electric field in *xoz*-plane at the frequencies *f* = 10 GHz and *f* = 15.5 GHz, respectively. In the simulation, the LCP plane wave is employed as the wave source, and the boundary conditions in *x-, y*- and z-directions are all set to be open add space. Observed from the figure, we can find that the transmitted waves are all efficiently refracted with the refraction angles *θ*_*t*_ ≈ 56° and 33° at the two frequencies.

Under the *x*-polarized wave normal incidence, the normalized polarization conversion transmission spectrum is simulated and the result is shown in Fig. 7(c). The *x* and *y* coordinates denote the frequency and refraction angle, respectively. The color in the figure labels the normalized polarization conversion transmittivity. It is found from the figure that the transmitted wave is decomposed into two beams of CP waves and refracted along opposite directions. The refraction angle versus frequency is well consistent with the case of the CP wave incidence as shown in Fig. 7(a). The distributions of the electric field *x*-component in *xoz*-plane for the *x*-polarized wave normal incidence at the frequencies *f* = 10 GHz and 15.5 GHz are illustrated in Fig. 7(d). The distributions of the electric field indicate that the transmitted waves are decomposed into two beams and efficiently refracted along opposite directions.

## Experiment verification

**Figure 8:**
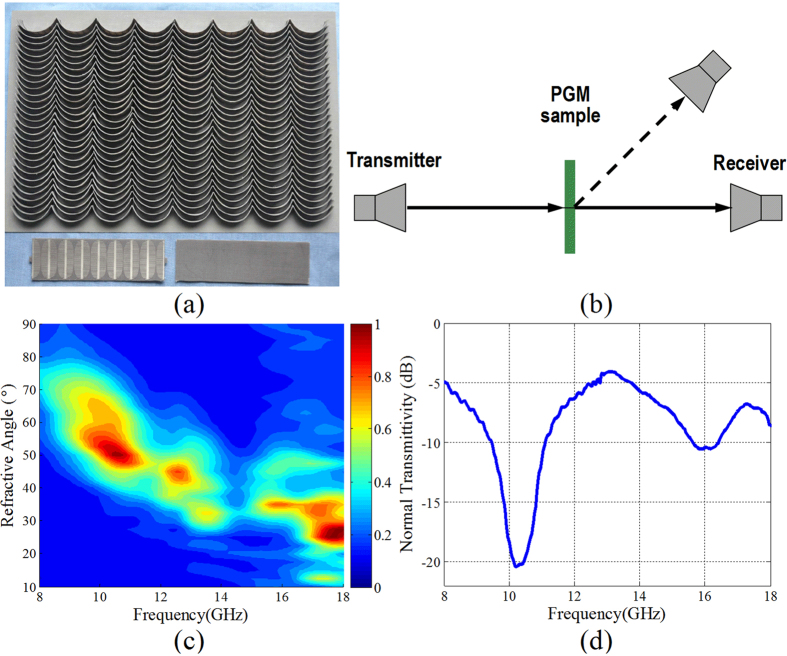
Experimental verification of the tri-band quasi-continuous PGM under LP wave normal incidence. (**a**) Photograph of the fabricated PGM sample consisting of a 30 × 8 array of super units. (**b**) Schematic diagram of the experimental measurement setup. (**c**) Measured normalized polarization conversion transmission spectrum for *x*-polarized wave normal incidence. (**d**) Measured normal transmittivity under *x*-polarized wave normal incidence.

In order to further verify the designed tri-band quasi-continuous PGM, the PGM sample with size of 300 mm × 300 mm was fabricated as shown in Fig. 8(a). The planar plasmonic structures for the super units of the PGM were fabricated using print circuit board (PCB) technique firstly. The final super unit was achieved by bending the fabricated planar plasmonic structures into the curved surface shown in Fig. 5. The measurements were performed in an anechoic chamber. The experimental measurement setup is given in Fig. 8(b), in which a revolving stage is employed. Two horn antennas with standard gain are fixed at two spiral arms of the revolving stage. One is used as a transmitter, and the other as a receiver. The *x*- and *y*-polarized waves can be transmitted or received by placing the short side of the horn antenna in its direction. The PGM sample is located at the center of the revolving stage. As for the measurement of the transmission spectrum, the transmitter is directly head upon the PGM sample, the receiver received the transmitted wave at different directions by rotating the spiral arm. Figure 8(c) gives the measured normalized transmission spectrum of the PGM sample under the *x*-polarized wave normal incidence. It is observed that the measured transmission spectrum of the PGM sample reveals good accordance with the simulated result given in Fig. 7 except for a broader beam width. This is mainly attributed to the limited distance between the sample and the receiver and a large aperture of the receiver. The angular resolution of the receiver is so large that the receiver can simultaneously receive the transmitted wave in a wider angle domain about 15°. In addition, the normal transmittivity under the *x*-polarized wave normal incidence is measured and the result is given in Fig. 8(d). In the figure, we can find that the normal transmittivity is less than −8 dB in the three frequency regions: [8.5 13.3]GHz, [15.3 16.2]GHz, and [17.0 18.0]GHz. This indicates that the incidence wave is highly transmitted and anomalously refracted.

## Conclusions

In summary, we proposed to achieve high-efficiency transmissive quasi-continuous PGM based on SSPP. The planar plasmonic structure consisting of corrugated metallic strips was designed to couple and mediate the SSPP. The SSPP on the plasmonic structure was studied by the dispersion relationship. Via aligning the SSPP based transmission and the free space wave propagation in two orthogonal directions, a high-efficiency tri-band CTC PCM was achieved using a planar plasmonic structure with modulated spatial distribution of strip length. The phase of the cross-polarization transmission under CP wave incidence was manipulated with many degrees of freedom by PB phase. The high-efficiency tri-band quasi-continuous PGM was realized by bending the planar plasmonic structure of the tri-band PCM into a curved surface according to the PB phase. Under the CP wave incidence, the highly transmitted wave was converted into the cross-polarized CP wave and then anomalously refracted in three frequency bands. Under the LP wave incidence, the transmitted wave was decomposed into two beams of CP waves with different polarizations and then anomalously refracted along opposite directions. Both the simulated and measured results demonstrated this high-efficiency quasi-continuous PGM.

## Methods

### Simulations

Electromagnetic simulations are performed using a commercially available software package, CST Microwave Studio. The dispersion relations are calculated using the Eigen-mode solver with periodic boundary conditions along the *x, y* and *z* directions. The amplitudes and phases of the transmission parameters for the PCM are simulated using the Frequency domain solver and the electric field distributions are monitored simultaneously. In the simulations, unit cell boundary conditions in the *x* and *y* directions are used, and open boundary conditions in the *z* direction. The normal transmittivity, the distribution of the electric field component together with the normalized transmission spectrum of the PGM are simulated using the Time Domain Solver. The plane wave is employed as the source in the simulations.

### Fabrication

The plasmonic structures are fabricated using the PCB photolithography. The commercial F4B dielectric substrates are employed as the dielectric layers and the 17-μm-thick copper films as the metal parts. The final super unit of the PGM was derived by bending the planar plasmonic structure into a curved shape.

### Measurements

The experimental measurements of the PGM are performed in a microwave anechoic chamber. For the measurement setup, a pair of horn antennas with standard gain faced upon each other. One works as a transmitter and the other as a receiver. The polarization of the transmitted wave or the received wave can be manipulated via rotating the horn antennas. The normal transmittivity is measured by placing the PGM sample at the middle of the two horn antennas. As for the measurement of the normalized transmission spectrum, the transmitter was directly facing the PGM sample, the transmitted waves at different directions was measured by rotating the receiver in-plane.

## Additional Information

**How to cite this article:** Li, Y. *et al*. High-efficiency tri-band quasi-continuous phase gradient metamaterials based on spoof surface plasmon polaritons. *Sci. Rep.* **7**, 40727; doi: 10.1038/srep40727 (2017).

**Publisher's note:** Springer Nature remains neutral with regard to jurisdictional claims in published maps and institutional affiliations.
